# Sea Buckthorn and Rosehip Oils with Chokeberry Extract to Prevent Hypercholesterolemia in Mice Caused by a High-Fat Diet In Vivo

**DOI:** 10.3390/nu12102941

**Published:** 2020-09-25

**Authors:** Lubov Tereshchuk, Kseniya Starovoytova, Olga Babich, Lyubov Dyshlyuk, Irina Sergeeva, Valery Pavsky, Svetlana Ivanova, Alexander Prosekov

**Affiliations:** 1 Vegetable Food Technology Department, Kemerovo State University, Krasnaya Street 6, 650043 Kemerovo, Russia; terechuk_l@mail.ru (L.T.); centol@mail.ru (K.S.); sergeeva.76@list.ru (I.S.); 2 Institute of Living Systems, Immanuel Kant Baltic Federal University, A. Nevskogo Street 14, 236016 Kaliningrad, Russia; olich.43@mail.ru; 3 Single Center of Collective Use, Innovation Park, Immanuel Kant Baltic Federal University, A. Nevskogo Street 14, 236016 Kaliningrad, Russia; 4 Natural Nutraceutical Biotesting Laboratory, Kemerovo State University, Krasnaya Street 6, 650043 Kemerovo, Russia; soldatovals1984@mail.ru; 5 Department of General Mathematics and Informatics, Kemerovo State University, Krasnaya Street, 6, 650043 Kemerovo, Russia; pavva46@mail.ru; 6 Laboratory of Biocatalysis, Kemerovo State University, Krasnaya Street 6, 650043 Kemerovo, Russia; a.prosekov@inbox.ru

**Keywords:** dietary supplement, sea buckthorn and rosehip oils, chokeberry, antioxidant activity, hypolipidemic, hepatoprotective effects

## Abstract

Dietary supplementation based on sea buckthorn and rosehip oils with added chokeberry extract was studied. We added the dietary supplement to the feed mixtures for laboratory animals. The possible toxicological effects and hypocholesterolemic, hepatoprotective activity of the dietary supplement in vivo were studied. After the observation period (6 weeks), no significant changes were found in the mass of organs and blood serum of laboratory animals (*p* > 0.05). However, there was a decrease in hypercholesterolemic indicators. Regular consumption of sea buckthorn and rosehip oils with added chokeberry extract (dietary supplement “ESB-1”) by laboratory animals inhibited the activity of liver enzymes and increased the antioxidant activity of blood serum (after the subcutaneous injection of sunflower oil/oil solution of carbon tetrachloride) but was not sufficient to bring them to physiological standards. The hypocholesterolemic and antioxidant properties of our dietary supplement already allow us to consider it a component of functional food products or a dietary supplement base. However, the full range of its biologically active properties, including the hepatoprotective function and regulation of metabolic disorders, has not been studied yet, which sets the direction of further research in vivo models and clinical practice to confirm its effectiveness in humans.

## 1. Introduction

In addition to oncological diseases, cardiovascular diseases, hepatitis, and diabetes are the main ones causing death or disability. Such diseases can often be prevented by maintaining a healthy lifestyle and sticking to a healthy diet [1,2,3]. Plant food components with antioxidant properties, especially the ones with phenolic compounds, are of particular interest. Phenolic-enriched diets have been found to help prevent a wide range of diseases and slow down the aging process [4,5,6]. In addition, hepatoprotective, antiproliferative and anti-obesity effects of phenolic compounds have been reported [7,8,9]. A high content of polyunsaturated fatty acids (PUFA), mainly vegetable oil and nuts, is a key component of a healthy diet to prevent heart diseases [10,11].

The creation of products that combine the effects of PUFA and antioxidant substances, while preserving natural benefits, is both interesting and relevant for scientists, doctors, and manufacturers. Bright-colored fruits are known to be rich in antioxidant substances [12,13,14]. If these fruits were also rich in polyunsaturated fatty acids, like those in fish oil [15], they would be perfect healthy products.

Sea buckthorn berries can be considered such a product, as their oil has antioxidant characteristics and the benefits of fatty acids. Sea buckthorn berries contain vitamins (A, B_1_, B_2_, B_3_, B_6_, C, E, К, etc.), as well as organic acids (up to 2.5%), quercetin, flavonoids, macro- and microelements, fatty oil (9% in the pulp, 12% in the kernels), which consists of triacylglycerols with saturated and unsaturated fatty acids (palmitooleic, oleic, linoleic, linolenic).

Previously, it was proved that sea buckthorn berries and their derivatives possess a wide range of beneficial characteristics: anti-inflammatory, antitumor, antioxidant, antiatherosclerotic, cholesterol-lowering, hypoglycemic, hepatoprotective, and antidiabetic effects [16,17,18]. There are reports that sea buckthorn products are safe foods with no serious side effects, except for an increase in blood glucose levels in rats at a significant rate of consumption (>100 mg/kg/day) of berries per day [19,20,21]. Both sea buckthorn berries and their derivatives are often considered as the basis of therapeutic and/or preventive food products [22,23].

Sea buckthorn oil extracted from both seeds and pulp is the main source of PUFA [24,25]: mature seeds contain 8–20% of oil, dried fruit pulp (pulp and peel)—about 20–25%, and fruit residue contains about 15–20% of oil after juice extraction [24,26]. These oils have a high concentration of lipophilic components, primarily unsaturated fatty acids in the form of triglycerides, phytosterols, and vitamins A and E [25,27,28,29]; they have a positive effect on human health, especially on the cardiovascular system [26,30].

Another promising raw material for functional products is rosehip, the fruit of which contains a large amount of vitamins (C, B2, A, K, P, and E) and fatty oil [31,32,33]. The oil content in rosehip seeds varies from 5 to 18% wt. and consists of a significant amount of unsaturated fatty acids [34,35]. Other significant groups of biologically active rosehip compounds are galactolipids and unsaturated fatty acids [36,37]. The fatty acid composition of rosehip oil is represented by stearic (up to 4%), palmitic (5%), oleic (up to 20%), linoleic (up to 50%), and linolenic acids (up to 40%). Lycopene makes rosehip and its products beneficial for the prevention of cardiovascular diseases and cancer [33,37,38,39,40,41,42]. Rosehip fruits have anti-inflammatory, antioxidant, antiproliferative, and antidiabetic properties [31,43,44,45,46,47,48].

Chokeberry (*Aronia melanocarpa*) is a rich source of polyphenols (including anthocyanins, flavonols, flavanols, proanthocyanidins, and phenolic acids), which determine the high biological activity of its berries [49,50]. Chokeberry fruits and their derivative products contain vitamins P, A, E, PP, and B-vitamins, microelements, amygdalin glycoside, and have great health potential, as they reduce the risk of metabolic syndrome development.

It has been confirmed (in vitro and in vivo) that chokeberry has beneficial effects on various diseases (dyslipidemia, hypertension, obesity, glucose metabolism disorders, pro-inflammatory conditions, and the risk of thrombosis) [51,52]. 

Mixtures of medicinal plants have been traditionally used since ancient times [53,54]. Mixtures may contain leaves, roots, shoots of plants, as well as their seeds, berries, fruit. It is important to follow the rules of preparation of mixtures, so that plant components reinforce each other’s actions and are safe to consume. Similar rules are applied to the development of dietary supplements. The aim of the study was to evaluate the effect of sea buckthorn oil, rosehip oil, and chokeberry extract combined on the manifestation of hypercholesterolemia in mice due to the predominance of fat in the diet.

## 2. Materials and Methods

### 2.1. Materials

“ESB-1” dietary supplementation (the patent application is under consideration at Rospatent http://www1.fips.ru) was the object of this study—a microemulsion (containing 100 g of 90.0% fat, PUFA ω-3 not less than 25.3%, PUFA ω-6 not less than 25.3%, omega-9 not less than 12.0%, essential phospholipids not less than 10.0%, α-tocopherol 17.9%, β-carotene 0.1%, rutin 3.3%). The supplement was obtained from the composition of vegetable oils of different fatty acid groups (linseed, rapeseed, sunflower, taken at the ratio of 50:20:30) using the fruit raw material derivate products (sea buckthorn (*Hippóphae rhamnoídes*)) and rosehip (*Rosa Cinnamomea*) at the ratio of 1:1, pre-dried to a moisture content of 12% and crushed to powder). Vegetable phospholipids (soy lecithin, 93% phospholipid content, Art life, Tomsk, Russia) and water extract of chokeberry (*Arónia melanocárpa*) were added to the mixture that was then thoroughly mixed. The content of active components in the “ESB-1” dietary supplement is shown in Table 1.

### 2.2. Feed Mixtures 

Feed mixtures were made up of components according to the recipes (Table 2), as described in our previous study [55,56]. Weighing was performed on laboratory scales (accuracy 0.01 g). Saccharose, AIN-93-VX vitamin mix, L-cysteine, choline bitartrate and corn starch were stirred in a mixer until a homogeneous mixture was obtained for 15 min at a rotation speed of 60 ± 5 rpm. Next, casein and a mixture of mineral salts AIN-93M were added, stirred for another 10 min, then corn starch dextrinate was added to the mixture and stirred for another 15 min. In the homogenizer, 2-tert-butylhydroquinone in soy oil and a ghee solution were added to the resulting mixture. According to the recipe, soybean oil (mixture No. 1) and cholesterol (mixture No. 2 and No. 3) were homogenized for 10 min. At the last stage, the “ESB 1” dietary supplement (mixture No. 3 and No. 7) was added and the components of the mixtures were homogenized for 10 min. The prepared mixtures were stored in sealed plastic containers for no more than 5 days at a temperature of (4 ± 2) °C.

### 2.3. Experimental Animals 

Male white rats (Wistar strain, age—4 months, weight—not less than 300 ± 1 g) were purchased from the Center for Genetic Resources of Laboratory Animals of the Institute of Cytology and Genetics the Siberian Branch of the Russian Academy of Sciences (Novosibirsk, Russia). The rats were given free access to the feed (granulated complete feed, BioPro, Russia) and drinking water. Prior to the experiment, the animals were quarantined for at least 2 weeks, as described in our previous study [55,56]. All rats were placed in polycarbonate cages (5 in each cage; at a temperature of 22 ± 2 °C; relative humidity of at least 60%). Each cage had drinkers, steel label holders, steel feed dividers, steel latticed lids with a feed recess, removable prefilters and HEPA (High Efficiency Particulate Air) air filters. The daily cycle of animals was 12 h of light and 12 h of darkness. The study was conducted in accordance with the Declaration of Helsinki; The BioEthical Committee of the Research Institute of Biotechnology of the Kemerovo State University approved the experimental protocol (Project identification code 1118/23.11.2018).

### 2.4. Experimental Design

Three days before the experiment (after 14 days of quarantine), all animals (each rat weighing up to 200 g) were randomly divided into groups. The control and experimental groups included 15 animals each. The animals were placed in individual ventilated cages. The air renewal rate in the system was not less than 15 m^3^/h. The cages were placed in a rack-shelf. 

During the study, the following parameters were maintained (air temperature −22 ± 2 °C, relative humidity—at least 60%, air velocity in the system—at least 15 m^3^/h, and the daily cycle of light and darkness—12 h). The animals were allowed drinking water ad libitum. Straw bedding, food and drinking water were changed daily. For animals weighing under 350 g, the daily portion of food weighed 25 g (basic nutrients) or 30 g for animals weighing over 350 g. At least once a week, experimental animals were weighed on laboratory scales (absolute error ±0.1 g) in a 5 dm^3^ plastic cup. All experiments on rats were conducted in the morning (in accordance with ethical principles for conducting painful experiments on animals and with the current guidelines for the care of laboratory animals).

During the entire study period, all animals were under constant observation. The experimental groups, the feeding regime, and the manipulations performed are presented in Table 3.

The animals were observed daily for 6 weeks. Animal behavior, their general condition, the nature and intensity of motor activity, the color of mucous membranes, skin, fur condition, the volume of feed and water consumed, tail position, mass of organs, biochemical and hematological parameters of blood, and blood serum (clinical signs of intoxication) were recorded during the intravital phase, as described in our previous study [55,56]. Twelve hours before the end, feed leftovers were removed from the cages. Animals from experimental groups were euthanized with carbon dioxide (feed rate—3.5 dm^3^/min; gas anesthesia system for laboratory animals, RWD Life Science, Shenzhen, Guangdong, P.R. China) depending on the animal’s body weight for 3 to 5 min. After the absence of respiratory movements was visually confirmed, the animals were removed from the euthanasia chamber. 

Blood sampling (5 cm^3^) was carried out using sterile syringes from the heart cavity after the 6-week experiment came to an end. Animals were put under anesthesia with ether vapors and fixed on the dissecting table with their belly up. Palpation on the left side of the chest determined the cardiac impulse position. The syringe needle was inserted 5 mm horizontally to the sternum to the point of cardiac impulse. After the euthanasia, organs (heart, lungs, liver, kidneys, spleen, thymus, adrenal glands) were taken for weighing. 

Animals from groups I–IV (Table 3) were tested for toxicological characteristics of “ESB-1” dietary supplement. Animals from groups VI and VII) were tested for the presence of hypocholesterolemic characteristics in “ESB-1” dietary supplement.

At the beginning of the experiment, group I animals were subjected to euthanasia with organ and blood sampling. Animals from groups VIII-X were manipulated as described in Table 3. Sterile refined, deodorized sunflower oil or an oil solution of carbon tetrachloride was injected into the withers subcutaneously with a 1.0 cm^3^ syringe at a dose of 1 mL/kg. In 24, 48 and 72 h after the administration of oil or oil solution, five animals of each group were euthanized with carbon dioxide and the biomaterial was taken for analysis.

### 2.5. Evaluation of Biochemical Parameters of Blood Serum

Using the solid-phase spectrophotometric method [57], the levels of cholesterol, triglycerides, high- and low-density lipoproteins (HDL and LDL), and unesterified fatty acids (UFA) were determined in serum samples using “Cholesterol” (BioSystems S.A., Barcelona, Spain), “Triglycerides” (BioSystems S.A., Barcelona, Spain), Cholesterol HDL Direct (BioSystems S.A., Barcelona, Spain), and 96-well Serum/Plasma Fatty Acid kit (ZenBio Inc., Morrisville, NC, USA) kits while following the manufacturers’ recommendations. The absorption of the solution was measured with a spectrophotometer (1 cm cuvette) at a wavelength of 500 nm, for UFA a wavelength of 540 nm and for lipoprotein wavelengths of 600 and 700 nm.

The Berthelot enzymatic colorimetric method was used to determine the urea concentration in the serum with a commercial kit (BioSystems S.A., Barcelona, Spain). The absorption of light produced by indiphenol is proportional to the urea content in the sample at a wavelength of 500–560 nm. 

A spectrophotometer with a thermostatic compartment (wavelength 340 nm, temperature 37 °C, quartz cuvettes 1 cm) was used to determine the activity of alanine aminotransferase (ALT) and aspartate aminotransferase (AST) in the samples of serum as described in our previous study [55,56] with Alanine aminotransferase ALT/GPT (BioSystems S.A., Barcelona, Spain) and Aspartate aminotransferase AST/GOT (BioSystems S.A., Barcelona, Spain) kits, following the manufacturer’s instructions. 

The RAPID N (Cube protein nitrogen analyzer is an analyzer for fast and absolutely safe determination of nitrogen (protein) in food) Cube protein nitrogen analyzer and Dumas method were used to determine total protein content in the blood serum. The Dumas method is based on measuring the thermal conductivity of molecular nitrogen produced after combustion of the sample in an oxygen atmosphere at a temperature of about 1000 °C, followed by a reduction of all the resulting nitrogen oxides with a reducing agent (copper).

Capillary electrophoresis (Kapel-105, Lumeks, Russia) was used to determine globulins and albumins in the serum.

The Popper method based on the Jaffe reaction with a commercial kit (BioSystems S.A., Barcelona, Spain) determined creatinine in the blood serum. 

Bilirubin in the serum of laboratory animals was determined by measuring bilirubin absorption using the spectrophotometric method (BioSystems S.A., Barcelona, Spain) at 440–460 nm.

Automated hematological analyzer ADVIA 60 (Siemens, Germany) was used to determine hematological parameters (hemoglobin level; number of red blood cells, white blood cells, platelets; hematocrit; average concentration of hemoglobin in red blood cells) in the serum samples.

### 2.6. Testing of Hepatoprotective Properties 

Spectrophotometrically, using Lactate dehydrogenase (LDH), Aspartate aminotransferase AST/GOT, and Alanine aminotransferase ALT/GPT (BioSystems S.A., Barcelona, Spain) commercial kits and following the manufacturer’s recommendations, the activity of LDH, AST, and ALT was determined in the serum. Optical density of the solutions was determined using a spectrophotometer with a thermostatic compartment (1-cm quartz cuvettes, temperature 37 °C, wavelength of 340 nm).

In serum and liver homogenates, the concentration of TBA-reactive products was determined using a non-absorbing microplate spectrophotometer (wavelength 535 and 572 nm); it was expressed in the equivalent of the concentration of malondialdehyde (MDA) [58,59].

### 2.7. Testing of Antioxidant Activity

Kinetic spectrophotometry (wavelength 734 nm, data collected at intervals of 1 min, duration 40 min) was used to determine the antioxidant activity of the dietary supplement to reduce the concentration of ABTS cationic radical in the blood serum; it was expressed through the equivalent concentration of the water-soluble analogue of vitamin E—Trolox [60].

Values determining the ability to absorb oxygen radicals were determined by the equation:

ORAC (oxygen radicals absorption capacity) = X·K·(S_sample_ − S_blank_)/(S_trolox_ − S_blank_),

where X is the volume of the sample (μ), K is the dilution coefficient of the sample, and S is the area under the fluorescence decay curve of the sample.

### 2.8. Statistical Analysis

Each experiment with the biomaterials was repeated three times. The data in the tables were expressed as means ± standard error of mean (SEM), and the data in the figures were represented as means ± standard error (SE). The homogeneity of the sampling effects was checked using Student’s *t*-test. The data were subjected to analysis of variance (ANOVA) using Statistica 10.0 (StatSoft Inc., 2007, Tulsa, Ok, USA). Differences between means were considered significant when the confidence interval was smaller than 5% (*p* ≤ 0.05). 

## 3. Results

The potential of plant-based components and the production of functional products by processing vegetables and fruits is well known and has been used by us multiple times [55,61,62,63,64,65]. Our works [56,66,67] present the results of studying bio-functional properties in vitro and the potential to increase physical endurance with help of pine nut processing products, including oil, in vivo. We went further and developed a dietary supplement based on sea buckthorn and rosehip oil. To enhance its biological activity, chokeberry extract was added. 

**Figure 1:**
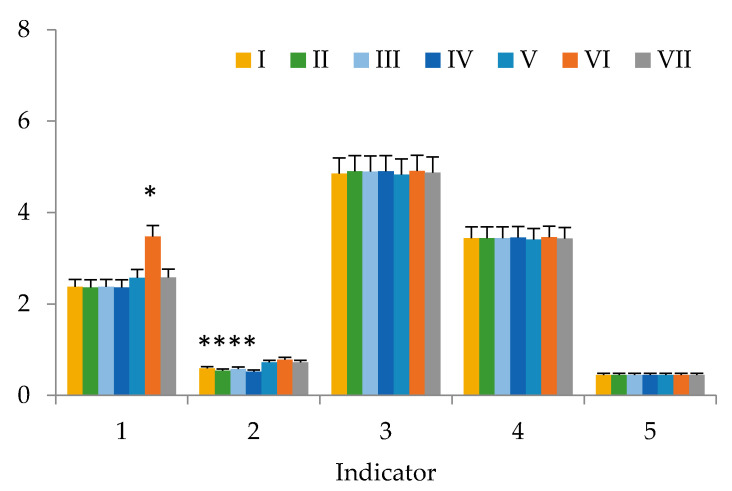
Biochemical and hematological indices of blood of animals from groups I–VII: 1—Cholesterol, mmol/L; 2—Triglycerides, mmol/L; 3—Bilirubin, μmol/L; 4—Number of leukocytes, g/L; 5—Hematocrit, L/L (bars in graphs indicate the SE of 15 replicates). Values with «*» do differ significantly (*p* > 0.05).

**Figure 2:**
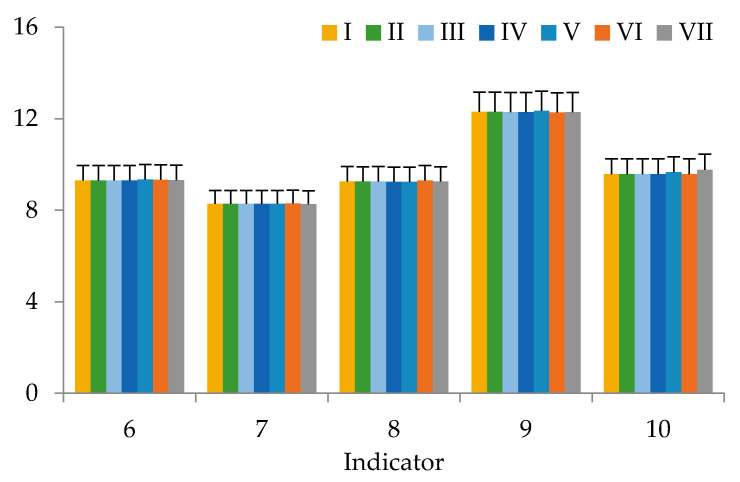
Biochemical and hematological indices of blood of animals from groups I–VII: 6—Urea, mmol/L; 7—ALT, U/L; 8—AST, U/L; 9—Globulins, g/L; 10—Red blood cell count, g/L; (bars in graphs indicate the SE of 15 replicates).

**Figure 3:**
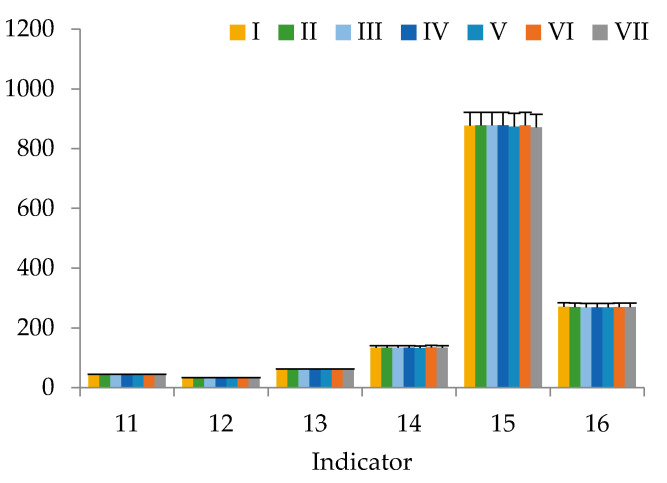
Biochemical and hematological indices of blood of animals from groups I–VII: 11—Total protein, g/L; 12—Albumin, g/L; 13—Creatinine, μmol/L; 14—Hemoglobin level, g/L; 15—Platelet count, g/L; 16—Average concentration hemoglobin in the erythrocyte, g/L (bars in graphs indicate the SE of 15 replicates).

This work presents the results of studying the potential of using our dietary supplement as a component of functional nutrition in vivo. There were no deaths or signs of intoxication in the in vivo study during the follow-up period, including acute toxicity during the first 24 h after the dietary supplement intake. There were no significant differences (*p* > 0.05) in body weight and height, skin and fur between the control group and the rats that were fed our dietary supplement. Under the influence of the dietary supplement, the behavior of animals and their intake of food did not change. After 6 weeks (Figure 1, Figure 2 and Figure 3), serum levels of laboratory animals remained unchanged regardless of their diet (*p* > 0.05, by LSD (LSD method—Least Square Difference method of statistics) post-hoc test), except for cholesterol and triglycerides. Their high content depended on the diet. Animals of group’s I–IV did not receive either animal fat or cholesterol in their diet, and animals of groups V–VII had animal fats (ghee) in combination with or without cholesterol in their diet. There were no significant differences in the weight of organs between the groups of animals. In all animal groups, the organ weight was as follows: heart 1.25 ± 0.06 g, lung 1.46 ± 0.06 g, liver 9.56 ± 0.47 g, kidney 3.12 ± 0.15 g, spleen 0.93 ± 0.05, thymus 0.42 ± 0.02, adrenal glands 0.11 ± 0.01 g. Toxicological studies [68] of the dietary supplement based on sea buckthorn and rosehip oils with added chokeberry extract confirmed its safety, since no toxic effect on the body of laboratory animals has been established.

The results of the study of blood samples of laboratory animals from groups I–VII show that, after 6 weeks, there were statistical significant increases in cholesterol and triglycerides in the blood serum. The triglyceride content (Figure 1) was statistically determined by the presence or absence of animal fats (ghee) in the diet. Cholesterol and triglyceride values in groups of animals taking a diet with a significant amount of animal fats (mixture No. 6) were significantly different from the control animals (*p* = 0.899 and *p* = 0.921, respectively). For laboratory animals consuming mixture No. 7 with a significant content of animal fats, no significant differences from the control group in regard to cholesterol were revealed, just like with mixture No. 6. 

Comparing these indicators in animals from Groups VI-VII, whose diets differed only in the presence or absence of a dietary supplement based on sea buckthorn and rosehip oils with added chokeberry extract, the positive effect of this supplement is observed. Apparently, the biologically active components of sea buckthorn and rosehip oils with added chokeberry extract in the supplement activated the gastrointestinal system function, leading to the splitting and processing of additional cholesterol administered into the body of laboratory animals according to the consumed formula.

Changes in the activity of liver enzyme indicators in the serum of laboratory animals from groups VIII–X after the administration of oil or an oil solution of carbon tetrachloride (Table 4) demonstrate significant liver damage. This is evidenced by an increase in the content of AST and ALT in the serum of the animals from the considered groups as compared to the control: from 1.2 to 3.5, from 2.2 to 5.9, 2.3 to 7.8 times and from 1.3 to 2.8, from 2.7 to 5.7, from 3.0 to 5.7 times, respectively, 24, 48 and 72 h after subcutaneous administration (Table 1). Animals from group IX had the highest content of liver enzymes; the introduction of oil solution of carbon tetrachloride without any intake of our dietary supplement led to the highest liver damage. Adding sea buckthorn and rosehip oils and chokeberry extract to the diet (group X) did not lead to AST and ALT values comparable to those in the control group but significantly minimized the negative impact of subcutaneous administration of solution of carbon tetrachloride on the liver. 

The performed manipulations have led to liver damage, which is out of question (groups VIII-IX). There were some positive changes in the animals from group VIII in 72 h after the manipulation; however, in this case, it seems that a longer rehabilitation period is needed to restore liver function. For group IX animals, negative effects accumulated throughout the entire observation period (72 h), which, it seems, cannot be neutralized by the body’s own protective forces. The results of group X indicate that the intake of sea buckthorn oil, rosehip oil, and chokeberry extract during the preparatory period allowed creating an internal protective barrier. It is possible that an increase in the rehabilitation period (more than 72 h) or an increase in the dosage of the consumed biologically active complex could provide complete recovery of liver functions, although additional research is required.

Antioxidant activity (Table 3) of the laboratory animal serum was dramatically reduced by the oil solution of carbon tetrachloride administered subcutaneously. Vegetable oil administration also led to a decrease in this indicator, but not so dramatically. The decrease in antioxidant activity in 72 h after administration reached 65% and 30% for oil solution of carbon tetrachloride and vegetable oil, respectively. For the group that took the dietary supplement with sea buckthorn and rosehip oils with added chokeberry extract, the decrease in antioxidant activity did not exceed 11%, almost corresponding to the indicators of the control group.

## 4. Discussion

We do not know any works that would study the properties of dietary supplements or products with similar formulations. There are works that study properties of the key components of our supplement separately. Sea buckthorn oil is known to have anti-inflammatory properties [69] and promote tissue regeneration [70]. Rosehip fruit extracts have been proven to have antioxidant, anti-inflammatory, immunomodulatory, anti-tumor, cardioprotective, antidiabetic, neuroprotective, and antimicrobial properties [71]. However, the hypolipidemic, hepatoprotective properties of sea buckthorn, rosehip oils and chokeberry extract, especially in the composition of bioactive supplements, have not been studied enough.

Our results are consistent with the results of [72]. For 60 days, sea buckthorn oil was added to the feed of white rabbits. One group was a control group, one group was fed sea buckthorn oil only, one—cholesterol diet (1 mL/day) only, and one more—cholesterol diet and sea buckthorn oil (after 30 days of high-cholesterol diet received sea buckthorn oil 1 mL/day for the next 30 days). Feeding sea buckthorn oil to common rabbits for 18 days resulted in a significant decrease in cholesterol, LDL, atherogenic index, and LDL/HDL ratio in the blood plasma. The cholesterol diet in rabbits decreased the acetylcholine-induced vasorelaxant activity, but was restored to normal values by the administration of sea buckthorn seed oil. The consumption of sea buckthorn oil after a high-cholesterol diet reduced both cholesterol and triglycerides, but did not lead to results of a pre-cholesterol diet. In our study, a similar effect is achieved in less time, apparently due to the synergistic effect of sea buckthorn and rosehip oils with the addition of chokeberry extract.

Hsu et al. [73] studied the protective effect of sea buckthorn seed oil on liver damage in mice caused by carbon tetrachloride (CCl_4_). It was found out that oral supplementation of 0.26 mg/kg to 2.6 mg/kg of sea buckthorn seed oil for eight weeks reduced the increased levels of ALT, AST, alkaline phosphatase (ALP), triglyceride (TG), and cholesterol by at least 13% in serum, which was induced by CCl_4_ (1 mL/kg) in mice. In our study, a single subcutaneous dose of CCl_4_ (1 mL/kg) was administered, which led to similar results in liver enzyme activity. Apparently, the hepatoprotective effect of our food supplement is mainly due to the presence of sea buckthorn oil, although the effect of rosehip oils and chokeberry extract cannot be underestimated. If there was an opportunity for a longer recovery of the liver by consuming our dietary supplement, the results would be comparable not only by quality but also by quantity (a decrease in the activity of liver enzymes by at least 50%).

Liu et al. [74,75,76,77] studied the properties and characteristics of flavonoids derived from rosehip. Antioxidant activity, hepatoprotective effect (liver damage caused by paracetamol or CCl_4_), protective effect in human umbilical vein endothelial cells have been proven in vitro. The mice were divided into seven groups that consumed or did not consume the rosehip flavonoid drug. After 7 days of consumption, CCl_4_ was injected subcutaneously to each mouse from groups 3–7, while groups 1–2 received olive oil. Mice that consumed the rosehip flavonoid drug received less liver damage, which confirmed the hepatoprotective effect of the drug. In the work of Valcheva-Kuzmanova et al. [78], the hepatoprotective effect of chokeberry juice was established (damage to the liver of rats by carbon tetrachloride). The administration of CCl_4_ increased the activity of AST and ALT in plasma, induced lipid peroxidation. Chokeberry juice reduced the manifestation of damage to the liver of rats and inhibited the increase in the activity of liver enzymes. The authors suggested that the possible mechanisms of this were determined by the fact that the antioxidant properties of the juice reduced CCl_4_ activity and activated protective mechanisms that restored damaged liver tissue. 

In our dietary supplement, the corresponding concentration of chokeberry is slightly lower, and the activity of its biologically active substances is certainly lower, because it is represented by an extract, but in combination with other active ingredients (sea buckthorn and rosehip), a similar positive effect on the liver of mice is observed. Apparently, the method used for obtaining oil from sea buckthorn and rosehip allows one not only to transfer biologically active substances from fruit to oil, but also to enhance the hepatoprotective activity of our biologically active food supplement.

Food products containing sea buckthorn oil are rich in PUFA, model lipid metabolism in the liver, and have pronounced hepatoprotective properties [79,80,81,82,83]. The dosage of our supplement’s active components in the diet of laboratory animals was not enough to bring the activity of liver enzymes and antioxidant activity of the blood serum to physiological norms. However, it determines the prospects for the synergistic action of biologically active components of natural fruits, which can become quite an alternative to synthetic substances. Additional research will expand the field of knowledge on this topic.

## 5. Conclusions

The functional properties of sea buckthorn oil, rosehip oil, and chokeberry extract are well known, and the study of a dietary supplement based on them showed that it has no less biologically active characteristics. Our study confirmed the effectiveness of “ESB 1” dietary supplement in reducing ALT and AST levels, reducing liver oxidative processes, and modulating antioxidant protection. The safety of experimental samples of dietary supplements based on sea buckthorn and rosehip oils with added chokeberry extract and their hypocholesterolemic and antioxidant properties have been proven, which suggests using sea buckthorn oil, rosehip oil, and chokeberry extract as a functional formula component of other foods, or the basis of dietary supplements. 

The possibility of using the formulated dietary supplement as a nutritional alternative to existing analogues used in medical practice for the treatment and prevention of lipid-carbohydrate metabolism disorders requires additional research. However, the full range of therapeutic and preventive effects, including hepatoprotective, of our dietary supplement based on sea buckthorn oil and rosehip oil with the addition of chokeberry extract have not been fully studied. The possible activity of the components of our dietary supplement in regulating lipid metabolism, reducing oxidative stress and liver inflammation suggests that it has a lipolytic effect when used by people with increased body weight, but this is a theme for future studies. 

## Figures and Tables

**Table 1:** Active substances in the “ESB-1” dietary supplement (per one 2.0 g capsule).

Active Substance	Content
PUFA ω-3, not less	380 mg
including α-linolenic acid, not less than	175 mg
PUFA ω-6, not less than	380 mg
including linoleic acid, not less than	265 m
γ-linolenic acid, not less than	110 mg
ω-9 (oleic acid), not less than	180 mg
essential phospholipids	150 mg
α-tocopherol	400 IU
β-carotene	2 mg
rutin	50 mg

ESB—Extract of Siberian Berries.

**Table 2:** Feed mixture recipes (g/kg of feed).

Ingredients	Feed Mixture						
	No. 1	No. 2	No. 3	No. 4	No. 5	No. 6	No. 7
Corn starch	510.7	509.7	508.6	506.5	456.5	458.7	454.5
Dextrinate corn starch	155.0	155.0	155.0	155.0	155.0	155.0	155.0
Casein	145.0	145.0	145.0	145.0	145.0	145.0	145.0
Saccharose	90.0	90.0	90.0	90.0	90.0	90.0	90.0
Soybean oil	50.0	50.0	50.0	50.0	10.0	10.0	10.0
Ghee	-	-	-	-	90.0	90.0	90.0
AIN-93M (mixture of mineral salts)	35.0	35.0	35.0	35.0	35.0	35.0	35.0
AIN-93-VX (vitamin mix)	10.0	10.0	10.0	10.0	10.0	10.0	10.0
L-cysteine	1.8	1.8	1.8	1.8	1.8	1.8	1.8
Choline bitartrate	2.5	2.5	2.5	2.5	2.5	2.5	2.5
Cholesterol	-	-	-	-	-	2.0	2.0
“ESB-1” dietary supplement	-	1.0	2.1	4.2	4.2	-	4.2
2-tert-butylhydroquinone in soy oil, mg	8.0	8.0	8.0	8.0	8.0	8.0	8.0

AIN-93-VX, Vitamin Mix and AIN-93M, Mixture of Mineral Salts (MP Biomedical LLC, Santa Ana, CA, USA) are components of the recommended diet for rats and mice in scientific research.

**Table 3:** Experimental design.

Group	Number of Animals	Subcutaneous Injection	Diet
I	15	-	No. 1
II	15	-	No. 2
III	15	-	No. 3
IV	15	-	No. 4
V	15	-	No. 5
VI	15	-	No. 6
VII	15	-	No. 7
VIII	15	Sterile refined and deodorized sunflower oil, 1 mL/kg of weight	No. 1
IX	15	Oil solution of carbon tetrachloride, 1 mL/kg of weight	No. 1
X	15	Oil solution of carbon tetrachloride, 1 mL/kg of weight	No. 4

**Table 4:** Indicators of oxidative processes in the liver and blood serum antioxidant activity of laboratory animals.

Duration of Incubation, after Subcutaneous Administration of Oil/Oil Solution of Carbon Tetrachloride, h	Groups			
	I	VIII	IX	X
**Lactate dehydrogenase (U/L)**				
Control	208.92 ± 1.92 ^a^	-	-	-
24	-	234.67 ± 1.94 ^a^	463.98 ± 3.02 ^a^	265.47 ± 1.92 ^a^
48	-	243.02 ± 1.93 ^a^	508.57 ± 2.97 ^a^	304.31 ± 1.92 ^a^
72	-	266.04 ± 2.00 ^a^	586.12 ± 3.17 ^a^	316.22 ± 1.92 ^a^
**Aspartate aminotransferase (U/L)**				
Control	9.24 ± 0.17 ^a^	-	-	-
24	-	11.03 ± 0.32 ^a^	32.76 ± 0.63 ^a^	15.06 ± 0.18 ^a^
48	-	41.97 ± 0.34 ^b^	54.22 ± 0.38 ^b^	20.04 ± 0.23 ^b^
72	-	21.34 ± 0.24 ^b^	72.18 ± 0.51 ^b^	23.11 ± 0.22 ^b^
**Alanine aminotransferase (U/L)**				
Control	8.36 ± 0.21 ^a^	-	-	-
24	-	10.51 ± 0.32 ^a^	23.18 ± 0.42 ^b^	12.23 ± 0.72 ^a^
48	-	47.46 ± 0.54 ^b^	42.44 ± 0.38 ^b^	22.46 ± 0.78 ^b^
72	-	25.84 ± 0.57 ^b^	47.36 ± 0.45 ^b^	25.47 ± 0.45 ^b^
**TBA-reactive products (umol/dm^3^ MDA)**				
Control	3.31 ± 0.16 ^a^	-	-	-
24	-	3.95 ± 0.21 ^b^	4.23 ± 0.21 ^b^	3.27 ± 0.17 ^a^
48	-	6.86 ± 0.33 ^b^	8.46 ± 0.43 ^b^	3.43 ± 0.17 ^a^
72	-	6.92 ± 0.34 ^b^	11.18 ± 0.57 ^b^	3.66 ± 0.17 ^a^
**Homogenates of liver (nmol MDA/g)**				
Control	41.7 ± 4.2 ^a^	-	-	-
24	-	50.0 ± 5.1 ^b^	53.4 ± 5.4 ^b^	40.8 ± 4.1 ^a^
48	-	58.7 ± 5.8 ^b^	65.0 ± 6.6 ^b^	44.3 ± 4.3 ^a^
72	-	63.7 ± 6.3 ^b^	78.7 ± 7.9 ^b^	47.1 ± 4.6 ^a^
**Antioxidant capacity (umol TE/umol)**				
Control	5.65 ± 0.56 ^a^	-	-	-
24	-	4.56 ± 0.55 ^a^	3.13 ± 0.32 ^b^	5.75 ± 0.57 ^a^
48	-	4.11 ± 0.51 ^a^	2.75 ± 0.27 ^b^	5.36 ± 0.54 ^a^
72	-	3.99 ± 0.51 ^a^	2.00 ± 0.22 ^b^	5.10 ± 0.50 ^a^

The data are expressed as mean ± SE (*n* = 15). Values followed by different letters in a line have considerable differences (*p* > 0.05) by LSD post-hoc test. I group—without subcutaneous administration; feed mixture No. 1; VIII group—subcutaneous oil administration; feed mixture No. 1; IX group—subcutaneous oil solution administration; feed mixture No. 1; X group—subcutaneous oil solution administration; feed mixture No. 4. MDA: malondialdehyde; LSD method – Least Square Difference method of statistics. TE: tocopherol equivalent.
